# The Consistency Between the Chinese Essential Medicines List and Treatment Guidelines—Taking Oncology Medicines as an Example

**DOI:** 10.3389/fpubh.2022.943994

**Published:** 2022-07-07

**Authors:** Luyan Cheng, Caiyun Li, Xuefang Zhang, Yongfa Chen, Jianzhou Yan

**Affiliations:** ^1^School of International Pharmaceutical Business, China Pharmaceutical University, Nanjing, China; ^2^The Research Center of National Drug Policy and Ecosystem, China Pharmaceutical University, Nanjing, China

**Keywords:** essential medicines list, treatment guidelines, consistency, matching degree, oncology medicine

## Abstract

The concepts of “essential medicine” and “national medicine policy” were first put forward for the first time at the World Health Assembly in 1975 in an effort to alleviate the problem of medicine unavailability in developing and poor countries. The essential medicine system in China has experienced three development stages since 1979, when the concept of essential medicines was first introduced, to actively respond to the call of the World Health Organization. Currently, the essential medicines list published in China is the national essential medicines list (2018 Edition). In this study, we examined the consistency between the essential medicines for treating seven cancers (liver cancer, breast cancer, esophageal cancer, lung cancer, colorectal cancer, gastric cancer, and leukemia) and the recommended medicines by cancer treatment guidelines to determine whether the essential medicines are of high quality for clinical needs. The results indicated that the degree of similarity between oncology medicines on the essential medicines list and oncology medicines recommended by guidelines was low, with the majority falling between 30 and 60%. Therefore, to improve the quality of essential medicines, it is necessary to further improve the matching degree. In addition, to further improve the consistency between the essential medicines list and treatment guidelines, the following suggestions are put forward in this paper: (1). Formulate universal treatment guidelines; (2). When selecting essential medicines, greater consideration should be given to those recommended in the guidelines; (3). The essential medicines list and treatment guidelines should be concurrently updated; (4). The cycle for updating the essential medicines list and treatment guidelines should be shortened.

## Introduction

The concepts of “essential medicines” and “national medicine policy” were first put forward at the World Health Assembly in 1975 (1), and they quickly became a component of global public health. The 1978 Almaty declaration recognized “the provision of essential medicines” as one of the eight elements of primary health care. The current WHO Expert Committee on the selection and use of essential medicines believe that “essential medicines” are provided based on the disease burden and safety, effectiveness and economy of medicines, to meet the essential medicines needs of the people. The objective of implementing the essential medicines policy is to provide sufficient quantity, an appropriate dosage form, and quality assurance when the public can afford it (2). WHO proposes the following procedure for selecting essential medicines: first, establish the standard treatment guide or “path” according to the disease spectrum. Then, a list of essential medicines is selected and developed based on the standard treatment guidelines or “path” (3).

To actively respond to the call of the World Health Organization, China's essential medicine system has undergone three development stages since the introduction of the concept of essential medicines in 1979: the establishment stage of the essential medicine system from 1979 to 2009; the formation and improvement of the essential medicine system from 2010 to 2017; and the essential medicine system that has entered a new stage of development from 2018 to date.

In September 2018, the general office of the State Council proposed in its opinions on improving the national essential medicine system that attention should be paid to clinical diagnosis and treatment guidelines and expert consensus when selecting essential medicines. Currently, China selects essential medicines by consulting experts. This selection method relies heavily on clinical medication experience and the subjective judgment of experts, which is not objective enough (4), and treatment guidelines that are closely related to actual clinical needs do not play a great role in the selection of essential medicines.

Treatment guidelines are normative documents formulated for the diagnosis and treatment of a disease based on a comprehensive understanding of clinical evidence and demonstration by peer experts, which are designed to assist doctors and patients in making appropriate medical care decisions for specific clinical conditions (5). The treatment guidelines pay more attention to evidence-based medical evidence such as randomized controlled trials and open clinical trials, and also refer to other levels of evidence, such as literature meta-analyses, making them more scientific and practical.

The formulation process of treatment guidelines is very time-consuming and requires a significant amount of human and financial resources, but countries must still establish their guidance system and update it on a timely basis (6) because treatment guidelines have numerous benefits for patients, doctors, medicine supply managers, and health policymakers. It can improve compliance and availability of medicines for patients, as well as reduce the occurrence of adverse reactions. With the cost-effective methods provided by the guidelines, doctors can make appropriate treatment decisions for specific clinical manifestations, making the diagnosis and treatment of diseases and medicine selection more scientific, standardized, and standard, thereby effectively promoting essential medicines policy and rational medicine use (7, 8). Medicine supply managers are now better able to estimate the demand for medicines and effectively control inventory. For policymakers, the treatment guidelines can be incorporated into the assessment criteria used to evaluate and compare the nursing quality of different medical institutions and doctors (9), ensuring that the treatment guidelines are of great value.

In the past, essential medicines were selected from a list of specified medicines. Currently, essential medicines are selected from a list of therapeutic medicines recommended by treatment guidelines, thus, the selection of essential medicines should be more closely aligned with treatment guidelines (10). The selection of essential medicines combined with treatment guidelines can better improve the quality of essential medicines and establish the needs of medical institutions for essential medicines, which is critical for the diagnosis, treatment, and medication of patients (11). If the matching degree of essential medicines list and treatment guidelines is low, it will reduce doctors' recognition of essential medicines and dampen their excitement for clinical use of essential medicines.

Molds (12) argues that treatment guidelines should be developed first, and the essential medicines list should be composed of the medicines recommended by the guidelines. Only when treatment guidelines and the essential medicines list are developed and used together, as opposed to as single, irrelevant, and possibly contradictory entities, can they have a positive impact on clinical practice (13). The treatment guideline connects the essential medicines list to clinical practice. To supply high-quality medicines for clinical needs, the essential medicines list must be extremely consistent with treatment guidelines. Zeng FD (8) and Feng JJ (14) believed that the selection and dynamic adjustment process of the essential medicines list should be coupled with the formulation of treatment guidelines, and the implementation of the essential medicine list should be combined with the training of treatment guidelines, to promote the improvement of China's essential medicine system and guide clinical rational medicine use. South Africa has established an essential medicine selection model based on clinical guidelines, with treatment guidelines as the core, first to formulate treatment guidelines, and then to formulate an essential medicine list that includes all the drugs recommended in the treatment guidelines (15).

There are still some issues with the clinical application of essential medicines in China. There are disparities, for instance, between essential medicines and the actual medicine demand of a grass-root clinic. Zhang BY (16) analyzed the consistency between the medicines of a neurology department in a hospital and the medicines recommended in relevant guidelines and found that the consistency between the medicines used in hospitals for common diseases and the first-line and second-line medicines listed based on evidence is different. Some of the medicines recommended for clinical by doctors in the guidelines are not on the essential medicines list, indicating that there may be a mismatch between the guidelines and the current essential medicines list. In particular, the dark events of tumor therapy revealed by Dr. Zhang Yu of the Peking University Third Hospital last year once again brought attention to the treatment turmoil in the field of tumors. Ma Jun, director of Harbin Institute of Hematology and tumor and chairman of the board of supervisors of the Chinese Society of Clinical Oncology (CSCO), also pointed out that non-standard treatment is a common problem in tumor treatment (17). Research and analysis of 182 patients with chemotherapy drug reasonableness (18) published by the Journal of North Pharmacy in 2019 found that 37.91% of the 182 cancer patients were administered chemotherapy drugs inappropriately. The data cited in “Rational use of oncology drugs in 174 lung cancer patients” (19), which was published in the 8th issue of Central South Pharmacy in 2020, revealed that 83.9% of 174 patients with primary lung cancer admitted to a hospital in 2019 exhibited an unreasonable drug use pattern. In only 28 (16.1%) medical records, the oncology medication treatment protocol is plausible. Therefore, to standardize the clinical diagnosis and treatment of tumors, it is critical to formulate high-quality guidelines and a list of essential medicines that matches the guidelines.

Currently, the evaluation of essential medicines focuses primarily on the examination of the safety, effectiveness, and cost-efficiency of essential medicines. The study on the guidelines focuses mostly on guideline interpretation and quality assessment. No article evaluates the quality of essential medicines based on the consistency between the essential medicines list and treatment guidelines. It is necessary to investigate whether there is a mismatch between the essential medicines list and treatment guidelines, and if so, what is the main manifestation of the problem, and how it may be adjusted and improved in the future. China is currently in a critical period of adjusting the essential medicines list. This paper will evaluate whether essential medicines are high-quality medicines for clinical needs from the perspective of the consistency between the oncology drugs in the national essential medicines list (2018 Edition) and the oncology drugs recommended in the treatment guidelines, and will attempt to improve the matching degree between the list and the guidelines through research, as well as improve the role of China's essential medicines list in guiding clinical rational medicine use and accessibility.

## Methods

Firstly, the relevant data on China's disease burden was identified. Using the Mortality, morbidity, and risk factors in China and its provinces, 1990–2017: a systematic analysis for the Global Burden of Disease Study 2017(20) published by the lancet in 2019, this article analyzed and compared the mortality, years of life loss (YLLs), years of disabled life (YLDs), disability-adjusted life years (DALYs), and other indicators in all provinces in China between 1990 and 2017. This article reveals that cancer is still a serious disease in China. In 2017, eight types of tumors accounted for the top 25 causes of death in YLLs, including lung cancer (third), liver cancer (fifth), gastric cancer (seventh), esophageal cancer (eleventh), colorectal cancer (fifteenth), breast cancer (twenty-first), leukemia (twenty-second), and brain cancer and central nervous system cancer (twenty-third).

Then, using databases such as yaozhi.com and dingxiangyuan medication assistant, we obtained the drug instructions and analyzed the indications of oncology drugs in the national essential medicines list (2018 Edition), and the specific tumors involved were sorted according to the number of medicines used to treat each specific tumor, from greater to lesser. In the 2018 version of the national essential medicines list, there are 35 oncology medicines. Sodium ISO and ondansetron are used as oncology auxiliary medicines. So the above-mentioned medicines were not included in this study. Therefore, this study has collated indications of 33 oncology medicines. Leukemia (17 kinds of essential medicines), breast cancer (16 kinds of essential medicines), lung cancer (13 kinds of essential medicines), esophageal cancer (10 kinds of essential medicines), and gastric cancer (8 kinds of essential medicines) are the five kinds of tumors for which a large number of medicines are available.

Therefore, combined with the number of medicines for each tumor in the essential medicines list and the disease burden, the guidelines for seven specific tumors and the essential medicines list were finally determined. These seven tumors included lung cancer, liver cancer, gastric cancer, esophageal cancer, colorectal cancer, breast cancer, and leukemia.

After identifying the type of tumor, it is vital to locate relevant guidelines, as there are few official guidelines for each disease in China, the majority of which are formulated by relevant organizations, resulting in a significant number of disease-related guidelines of varying quality. Therefore, we formulated relevant standards to screen the guidelines, The specific screening criteria were as follows: (1) unit qualification: it must be a government department, an authoritative discipline association, or an organization with significant influence, such as the Chinese Medical Association, the National Health Commission of the people's Republic of China, the Chinese Medical Doctor Association, the Chinese Society of Clinical Oncology, etc., (21), to ensure the quality of guidelines; (2) Country: issued by the main body of China; (3) Release time: 2015–2022 or the most recent version of the guideline outside of this window, with the most recent version preferred; (4) Text type: the official guide is preferred. In the absence of a guide or if the publication date of the guide is too soon, the expert consensus is selected as appropriate (21); (5) Content of the guide: includes the main contents of tumor medicine therapy.

The essential medicines list and the guidelines will be compared from two perspectives following the screening of the guidelines: (1) compare the consistency between specific essential medicines and medicines recommended by the guidelines published in 2018 and before; (2) compare the consistency between specific essential medicines and medicines recommended by the guidelines published after 2018, which is quantified by the degree of matching. The matching degree formula is: matching degree = A / B ^*^ 100%, where A represents the number of medicines not only in the essential medicines list, but also in the guideline-recommended medicines, and B represents the number of drugs recommended in the first line of the guide or the number of medicines recommended in the guideline (when the recommendation level is not specified in the guideline).

The author carried out an expert examination into the problem of matching degree zoning to divide the matching degree into levels. Considering the widespread application of the Delphi method in a variety of disciplines and the varying degrees of methodological advancement, it is evident that this method is maturing. At the same time, to fully express the opinions of experts without being influenced by authoritative views, the Delphi method was used to synthesize the opinions of numerous experts in this survey. Delphi method involves the anonymous administration of many rounds of questionnaires to experts. Experts can think deeply about the survey problems and fully express their opinions in each round of the survey without worrying about the opinions of other experts. Multiple rounds of surveying will yield convergent expert opinions, with each survey feeding back the survey results from the preceding round to experts. The Delphi method has the advantages of anonymity and multiple rounds of investigation, which can increase the objectivity and reliability of the investigation's findings.

According to the content of this paper's research, 25 experts from medical and pharmaceutical colleges, institutions, departments, and enterprises across the country who have studied or are well-versed in essential medicines, treatment guidelines, and tumor clinical treatment were invited to conduct the survey. The analysis of expert authority degree evaluates the expert authority coefficient (Cr), the basis of expert judgment on matching degree zoning (Ca) and the expert's familiarity with essential drugs and guidelines (Cs). Cr = (Ca + Cs) / 2. The research result of Cr ≥ 0.7 is acceptable. The results of expert authority analysis show that in the first round, Cs was 0.80, Ca was 0.90 and Cr was 0.85; In the second round, Cs was 0.78, Ca was 0.91 and Cr was 0.85; In the third round, Cs was 0.82, Ca was 0.92, Cr was 0.87, and all three rounds' Cr was > 0.7, which was highly authoritative.

The survey involved three rounds of correspondence. In the first round of correspondence, experts received a summary of the matching degree zoning based on a literature review, an explanatory letter, and relevant background information. Then we consulted the experts whether it is necessary to partition the matching degree and score its importance (5-point system) and how to divide the matching degree into several levels. In the first round, 25 experts were consulted and 25 questionnaires were collected. The enthusiasm coefficient of experts was 100%. Through the analysis of the questionnaire, it is found that most experts believe that the matching degree needs to be divided. The coefficient of variation (CV) of the index of matching degree division is 0.11, indicating that the expert opinions are relatively coordinated. On the specific level of matching degree, 23 experts suggested that the matching degree be divided into five levels and two experts suggested that the matching degree be divided into four levels. Therefore, we decided to divide the matching degree into five levels. There are some differences in expert opinions on the division of specific levels. In the second round of correspondence, experts were provided with the modified matching degree zoning based on the opinions of the first round of experts and consulted again. In the second round, 25 experts were consulted, 24 questionnaires were recovered, and the enthusiasm coefficient of experts was 96%. After sorting out the questionnaire, it is found that the expert opinions on the division of most levels are closer, and there are differences between the division of the third level and the fourth level. Therefore, in the third round of correspondence, the matching degree obtained in the second round was fed back to the experts in layers, and the division standards of the third and fourth levels were further consulted. Similarly, 25 experts in the first round were consulted, and 24 questionnaires were collected. The expert enthusiasm coefficient was 96%. In this round of consultation, the opinions of experts tended to be consistent, and finally the matching degree zoning was determined: those with a matching degree of <30% were considered extremely mismatched, 31–50% were considered mismatched, 51–70% were considered more matched, 71– 80% were considered relatively matched, and 81–100% were considered highly matched.

## Results

### Liver Cancer

In China, liver cancer is one of the most prevalent malignant tumors. It has the fourth-highest incidence and second-highest mortality (20, 22). The essential medicines list (2018 edition) includes five medicines for the treatment of liver cancer. After screening, two guidelines published in 2018 and before are available for comparison: Standard for diagnosis and treatment of primary liver cancer (2017 edition) and Guidelines for diagnosis and treatment of primary liver cancer (2018 edition). The two guidelines recommend 4 first-line medicines, including 1 essential medicine for the treatment of liver cancer. The matching degree is 25.00%. Two guidelines published after 2018 are available for comparison: standard for the diagnosis and treatment of primary hepatic carcinoma (2019 edition) and the guideline for the radiotherapy of hepatocellular carcinoma in China (2020 edition). The two guidelines recommend 6 first-line medicines, including 1 essential medicine for the treatment of liver cancer. The matching degree is 16.67% (Table 1).

**Table 1:** Medicines in the list and guidelines for the treatment of liver cancer.

**Medicines**	**Essential medicines list** **(2018 edition)**	**Standard for diagnosis** **and treatment of primary** **liver cancer (2017 edition)**	**Guidelines for diagnosis** **and treatment of primary** **liver cancer (2018 edition)**	**Standard for diagnosis** **and treatment of primary** **hepatic carcinoma** **(2019 edition)**	**Radiotherapy guidelines** **for primary** **hepatocellular carcinoma** **in China (2020 edition)**
Oxaliplatin	✓	✓	✓	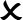	✓
Fluorouracil	✓	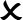	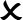	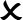	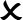
Arsenite	✓	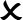	✓	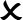	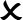
Doxorubicin	✓	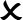	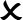	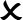	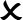
Pingyangmycin	✓	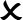	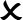	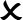	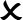
Sorafenib	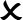	✓	✓	✓	✓
Cangvatinib	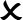	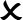	✓	✓	✓
Donafenib	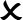	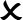	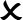	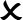	✓
Atilizumab	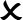	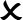	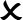	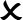	✓
Bevacizumab	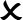	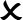	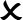	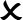	✓

### Breast Cancer

China ranks top in the incidence of female malignant tumors with a prevalence of about 3% for breast cancer (23). There are 16 breast cancer medicines on the essential medicines list (2018 edition). After screening, three guidelines published in 2018 and before are available for comparison: Chinese Advanced Breast Cancer Consensus Guideline (CABC 2015), Guidelines for breast cancer diagnosis and treatment (2017 edition) and breast cancer diagnosis and treatment guideline (2018.V1). The three guidelines recommend 27 first-line medicines, including 13 essential medicine for the treatment of breast cancer. The matching degree is 48.15%. Four guidelines published after 2018 are available for comparison: Chinese Advanced Breast Cancer Consensus Guideline 2020 (CABC3), breast cancer diagnosis and treatment guideline (2022), Guidelines for clinical diagnosis and treatment of advanced breast cancer in China (2020 edition), and the Guidelines for breast cancer diagnosis and treatment (2019 edition). The four guidelines recommend 21 first-line medicines, including 12 essential medicine for the treatment of breast cancer. The matching degree is 57.14% (Table 2).

**Table 2:** Medicines in the list and guidelines for the treatment of breast cancer.

**Medicines**	**Essential** **medicines list** **(2018 Edition)**	**Chinese Advanced** **Breast Cancer** **Consensus Guideline** **(CABC 2015)**	**Guidelines for breast** **cancer diagnosis** **and treatment (2017** **edition)**	**Breast cancer** **diagnosis and** **treatment guideline** **(2018.V1)**	**Guidelines for breast** **cancer diagnosis** **and treatment (2019** **edition)**	**Chinese Advanced** **Breast Cancer** **Consensus Guideline** **2020 (CABC3)**	**Breast cancer** **diagnosis and** **treatment** **guideline (2022)**	**Guidelines for clinical** **diagnosis and treatment** **of advanced breast** **cancer in China (2020** **edition)**
cyclophosphamide	✓	✓	✓	✓	✓	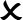	✓	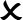
Gemcitabine	✓	✓	✓	✓	✓	✓	✓	✓
Doxorubicin	✓	✓	✓	✓	✓	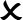	✓	✓
Paclitaxel	✓	✓	✓	✓	✓	✓	✓	✓
Tamoxifen	✓	✓	✓	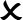	✓	✓	✓	✓
Letrozole	✓	✓	✓	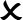	✓	✓	✓	✓
Trastuzumab	✓	✓	✓	✓	✓	✓	✓	✓
Capecitabine	✓	✓	✓	✓	✓	✓	✓	✓
Cisplatin	✓	✓	✓	✓	✓	✓	✓	✓
carboplatin	✓	✓	✓	✓	✓	✓	✓	✓
Ifosfamide	✓	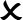	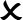	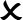	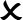	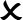	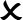	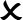
methotrexate	✓	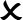	✓	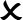	✓	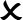	✓	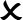
Fluorouracil	✓	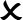	✓	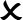	✓	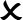	✓	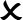
Pingyangmycin	✓	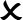	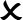	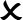	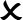	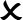	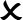	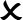
vincristine	✓	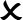	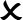	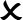	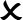	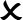	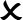	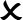
Etoposide	✓	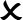	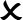	✓	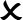	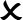	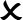	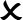
Fulvestrant	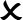	✓	✓	✓	✓	✓	✓	✓
Pertuzumab	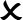	✓	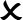	✓	✓	✓	✓	✓
Changchun Ruibin	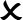	✓	✓	✓	✓	✓	✓	✓
Pyrroltinib	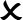	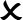	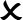	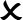	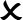	✓	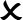	✓
Docetaxel	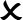	✓	✓	✓	✓	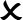	✓	✓
Piperacilli	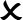	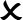	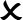	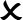	✓	✓	✓	✓
Torremifen	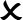	✓	✓	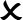	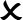	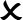	✓	✓
Epirubicin	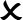	✓	✓	✓	✓	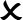	✓	✓
Doxorubicin liposome	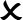	✓	✓	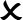	✓	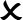	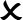	✓
Exemestane	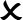	✓	✓	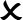	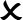	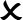	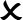	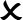
Anastrozole	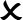	✓	✓	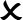	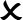	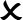	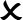	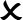
Everolimus	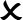	✓	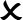	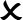	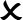	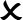	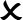	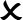
Lapatinib	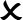	✓	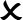	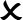	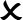	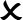	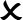	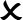
Paclitaxel (Albumin Bound)	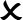	✓	✓	✓	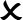	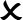	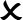	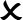
Eribulin	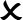	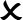	✓	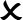	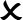	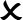	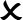	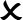
Pirarubicin	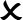	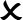	✓	✓	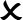	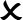	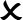	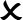

### Esophageal Cancer

Esophageal cancer is a high-risk malignancy in China. Its incidence and mortality are sixth and fourth respectively (24). There are 10 types of medicines for the treatment of esophageal cancer in the essential medicines list (2018 edition). After screening, one guideline published in 2018 is available for comparison: Standard for diagnosis and treatment of esophageal cancer (2018 edition). The guideline recommend 11 first-line medicines, including 7 essential medicine for the treatment of esophageal cancer. The matching degree is 63.64%. Two guidelines published after 2018 are available for comparison: the guidelines for diagnosis and treatment of esophageal cancer (2022 edition) and the Chinese guidelines for radiotherapy of esophageal cancer (2019 edition). The two guidelines recommend 16 medicines, including 8 essential medicine for the treatment of esophageal cancer. The matching degree is 50.00% (Table 3).

**Table 3:** Medicines in the list and guidelines for the treatment of esophageal cancer.

**Medicines**	**Essential medicines list** **(2018 edition)**	**Standard for diagnosis and** **treatment of esophageal** **cancer (2018 edition)**	**Guidelines for diagnosis and** **treatment of esophageal** **cancer (2022 edition)**	**The Chinese guidelines for** **radiotherapy of esophageal** **cancer (2019 edition)**
Paclitaxel	✓	✓	✓	✓
Fluorouracil	✓	✓	✓	✓
Cisplatin	✓	✓	✓	✓
Carboplatin	✓	✓	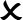	✓
Capecitabine	✓	✓	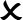	✓
Oxaliplatin	✓	✓	✓	✓
Calcium folinate	✓	✓	✓	✓
Trastuzumab	✓	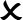	✓	✓
Tigio	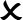	✓	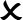	✓
Epirubicin	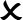	✓	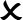	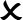
Nedaplatin	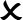	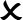	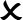	✓
Changchun Ruibin	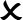	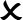	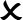	✓
Etoposide	✓	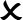	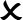	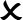
Pingyangmycin	✓	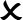	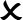	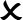
Docetaxel	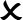	✓	✓	✓
Irinotecan	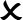	✓	✓	✓
Pembrolizumab	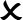	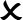	✓	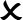
Nivolumab	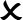	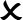	✓	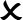
Camrelizumab	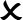	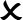	✓	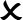

### Lung Cancer

Lung cancer has the highest incidence and mortality rate among malignant tumors in China (25). The national essential medicines list (2018 Edition) contains 13 types of essential medicines for the treatment of lung cancer. After screening, three guidelines published in 2018 and before are available for comparison: Expert Consensus on Diagnosis and Treatment of Advanced Primary Lung Cancer in China (2016 Edition), Guidelines for diagnosis and treatment of primary lung cancer (2018 edition) and Primary Lung Cancer Diagnosis and Treatment Standards (2018 edition). The guidelines recommend 21 first-line medicines, including 8 essential medicine for the treatment of lung cancer. The matching degree is 38.10%. Three guidelines published after 2018 are available for comparison: the clinical practice guideline for stage IV primary lung cancer in China (2021 version), the Chinese medical association guideline for clinical diagnosis and treatment of lung cancer (2019 edition), and the Guidelines for diagnosis and treatment of primary lung cancer (2022 edition). The three guidelines recommend 28 medicines, including 8 essential medicine for the treatment of lung cancer. The matching degree is 28.57% (Table 4).

**Table 4:** Medicines in the list and guidelines for the treatment of lung cancer.

**Medicines**	**Essential medicines** **list (2018 edition)**	**Expert Consensus on** **Diagnosis and Treatment** **of Advanced Primary** **Lung Cancer in China** **(2016 edition)**	**Guidelines for** **diagnosis and** **treatment of primary** **lung cancer (2018** **edition)**	**Primary Lung Cancer** **Diagnosis and Treatment** **Standards (2018 edition)**	**Chinese medical** **association guideline** **for clinical diagnosis** **and treatment of lung** **cancer (2019 edition)**	**Clinacal practice** **guideline for stage IV** **primary lung cancer** **in China (2021** **version)**	**Guidelines for diagnosis** **and treatment of primary** **lung cancer (2022 edition)**
Gemcitabine	✓	✓	✓	✓	✓	✓	✓
Etoposide	✓	✓	✓	✓	✓	✓	✓
Paclitaxel	✓	✓	✓	✓	✓	✓	✓
Cisplatin	✓	✓	✓	✓	✓	✓	✓
carboplatin	✓	✓	✓	✓	✓	✓	✓
Gefitinib	✓	✓	✓	✓	✓	✓	✓
Ektinib	✓	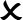	✓	✓	✓	✓	✓
Pemetrexed	✓	✓	✓	✓	✓	✓	✓
Erlotinib	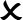	✓	✓	✓	✓	✓	✓
Afatinib	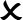	✓	✓	✓	✓	✓	✓
Daktinib	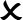	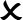	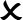	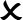	✓	✓	✓
Ositinib	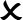	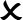	✓	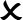	✓	✓	✓
Aletinib	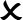	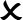	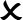	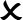	✓	✓	✓
Kezotinib	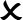	✓	✓	✓	✓	✓	✓
Bevacizumab	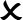	✓	✓	✓	✓	✓	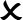
Pabolizumab	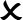	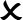	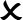	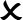	✓	✓	✓
Albumin paclitaxel	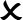	✓	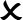	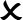	✓	✓	✓
Carrelizumab	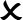	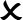	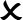	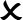	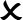	✓	✓
Changchun Ruibin	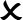	✓	✓	✓	✓	✓	✓
Docetaxel	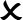	✓	✓	✓	✓	✓	✓
Irinotecan	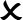	✓	✓	✓	✓	✓	✓
Atzumab	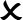	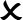	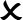	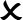	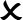	✓	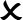
Lobaplatin	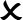	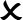	✓	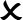	✓	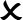	✓
Nedaplatin	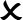	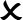	✓	✓	✓	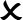	✓
cyclophosphamide	✓	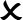	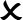	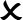	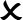	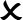	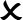
vincristine	✓	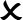	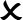	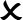	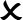	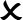	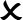
Ifosfamide	✓	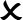	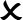	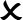	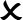	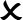	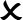
methotrexate	✓	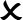	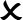	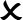	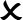	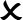	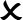
Doxorubicin	✓	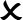	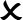	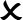	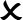	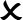	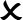
Tigio	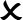	✓	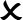	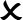	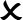	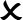	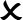
Topotecan	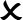	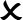	✓	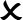	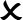	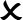	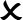
Tislelizumab	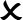	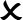	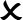	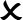	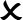	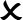	✓
Sintilimab	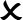	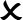	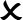	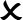	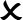	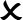	✓
Atezolizumab	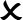	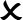	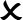	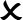	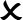	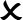	✓
Ceritinib	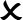	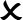	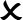	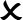	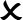	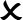	✓

### Colorectal Cancer

The incidence and mortality of colorectal cancer have been on the rise in China. China's 2018 cancer statistics revealed that the incidence and mortality of colorectal cancer in China ranked third and fifth among all malignant tumors, respectively (26). On the essential medicines list (2018 edition), there are 5 types of medicines for the treatment of colorectal cancer. After screening, two guidelines published in 2018 and before are available for comparison: China Colorectal Cancer Diagnosis and Treatment Standards (2017 edition) and Guidelines for the diagnosis and treatment of colorectal cancer (2018.V1). The guidelines recommend 10 first-line medicines, including 4 essential medicine for the treatment of colorectal cancer. The matching degree is 40.00%. Two guidelines published after 2018 are available for comparison: the Chinese protocol of diagnosis and treatment of colorectal cancer (2020 edition) and the guidelines for the diagnosis and treatment of colorectal cancer (2019 edition). The two guidelines recommend 10 medicines, including 3 essential medicine for the treatment of colorectal cancer. The matching degree is 30% (Table 5).

**Table 5:** Medicines in the list and guidelines for the treatment of colorectal cancer.

**Medicines**	**Essential medicines list** **(2018 edition)**	**China Colorectal Cancer** **Diagnosis and Treatment** **Standards (2017 edition)**	**Guidelines for the** **diagnosis and treatment** **of colorectal cancer** **(2018.V1)**	**guidelines for the** **diagnosis and treatment** **of colorectal cancer** **(2019 edition)**	**Chinese protocol of** **diagnosis and treatment** **of colorectal cancer** **(2020 edition)**
Fluorouracil	✓	✓	✓	✓	✓
Oxaliplatin	✓	✓	✓	✓	✓
Capecitabine	✓	✓	✓	✓	✓
Cetuximab	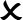	✓	✓	✓	✓
Bevacizumab	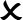	✓	✓	✓	✓
vincristine	✓	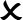	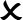	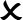	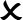
Calcium folinate	✓	✓	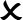	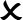	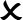
Irinotecan	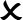	✓	✓	✓	✓
Aldehyde hydrofolate	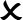	✓	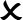	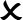	✓
Letitrexed	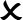	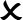	✓	✓	✓
Regofini	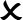	✓	✓	✓	✓
Furaquintinib	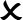	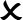	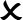	✓	✓

### Gastric Cancer

The incidence of stomach cancer in China is second only to lung cancer, while mortality ranks third (27). There are 8 kinds of essential medicines for the treatment of gastric cancer in the national essential medicines list (2018 edition). After screening, two guidelines published in 2018 are available for comparison: Standard for diagnosis and treatment of gastric cancer (2018 edition) and Guidelines for diagnosis and treatment of gastric cancer (2018 edition). The guidelines recommend 13 first-line medicines, including 7 essential medicine for the treatment of gastric cancer. The matching degree is 53.85%. One guideline published after 2018 is available for comparison: the guidelines for the diagnosis and treatment of gastric cancer (2022 edition). The guideline recommend 13 medicines, including 7 essential medicine for the treatment of gastric cancer. The matching degree is 53.85% (Table 6).

**Table 6:** Medicines in the list and guidelines for the treatment of gastric cancer.

**Medicines**	**Essential medicines list** **(2018 edition)**	**Standard for the diagnosis** **and treatment of gastric** **cancer (2018 edition)**	**Guidelines for diagnosis and** **treatment of gastric cancer** **(2018 edition)**	**the guidelines for the** **diagnosis and treatment of** **gastric cancer (2022 edition)**
Fluorouracil	✓	✓	✓	✓
Calcium folinate	✓	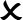	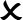	✓
Capecitabine	✓	✓	✓	✓
Cisplatin	✓	✓	✓	✓
Carboplatin	✓	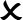	✓	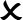
Oxaliplatin	✓	✓	✓	✓
Paclitaxel	✓	✓	✓	✓
Trastuzumab	✓	✓	✓	✓
Tigio	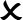	✓	✓	✓
Epirubicin	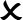	✓	✓	✓
Docetaxel	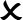	✓	✓	✓
Albumin paclitaxel	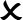	✓	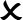	✓
Irinotecan	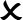	✓	✓	✓
Simustine	✓	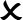	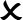	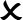
Apatinib	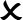	✓	✓	✓

### Leukemia

Leukemia is one of the most common malignant blood system tumors, and its mortality rate is high. The 2016 annual report on cancer registration in China revealed that leukemia incidence ranked 13^th^ and mortality ranked 10^th^ in the national cancer registration areas (28). There are 17 medicines for the treatment of leukemia in the essential medicines list (2018 edition), but the Compound Huangdai tablets and methylprednisolone are not classified as oncology drugs. After screening, two guidelines published in 2018 are available for comparison: Guidelines for the diagnosis and treatment of acute promyelocytic leukemia in China (2018 edition) and Guidelines for the diagnosis and treatment of chronic lymphocytic leukemia/small lymphocytic lymphoma in China (2018 edition). The guidelines recommend 15 first-line medicines, including 10 essential medicine for the treatment of leukemia. The matching degree is 66.67%. Two guidelines published after 2018 is available for comparison: guidelines for the diagnosis and treatment of chronic lymphocytic leukemia-small lymphocytic lymphoma (2022 edition) and guidelines for the diagnosis and treatment of chronic myeloid leukemia (2022 edition). The guidelines recommend 15 medicines, including 4 essential medicine for the treatment of gastric cancer. The matching degree is 26.67% (Table 7).

**Table 7:** Medicines in the list and guidelines for the treatment of leukemia.

**Medicines**	**Essential medicines list** **(2018 edition)**	**Guidelines for the diagnosis and** **treatment of acute promyelocytic** **leukemia in China (2018 edition)**	**The guidelines for the diagnosis** **and treatment of chronic** **lymphocytic leukemia / small** **lymphocytic lymphoma in China** **(2018 edition)**	**Guidelines for the diagnosis and** **treatment of chronic lymphocytic** **leukemia-small lymphocytic** **lymphoma (2022 edition)**	**Guidelines for the diagnosis and** **treatment of chronic** **myelogenous leukemia (2022** **edition)**
Arsenite	✓	✓	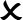	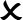	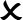
Hydroxyurea	✓	✓	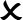	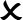	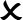
Cytarabine	✓	✓	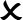	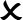	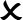
cyclophosphamide	✓	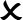	✓	✓	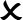
Compound Huangdai tablets	✓	✓	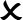	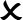	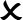
Fludarabine	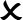	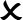	✓	✓	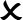
Rituximab	✓	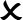	✓	✓	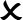
Bendamostine	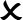	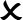	✓	✓	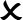
Nitrogen mustard phenylbutyrate	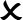	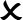	✓	✓	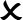
Ibutinib	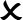	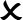	✓	✓	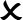
Methylprednisolone	✓	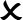	✓	✓	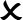
Oxaliplatin	✓	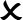	✓	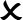	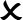
Retinoic acid	✓	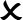	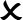	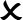	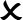
Daunorubicin	✓	✓	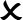	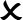	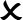
vincristine	✓	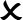	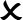	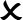	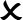
Mercaptopurine	✓	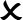	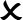	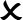	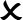
Homoharringtonine	✓	✓	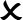	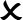	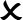
methotrexate	✓	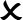	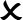	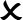	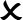
Doxorubicin	✓	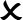	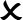	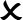	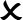
Asparaginase	✓	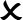	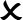	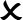	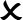
Imatinib	✓	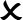	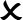	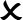	✓
Bai Xiaoan	✓	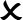	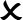	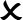	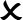
Lenalidomide	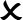	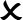	✓	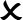	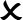
zanubrutinib	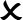	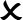	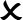	✓	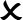
Orelabrutinib	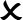	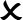	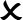	✓	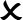
Venetoclax	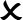	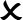	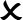	✓	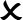
obinutuzumab	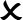	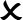	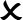	✓	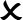
Nilotinib	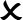	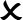	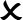	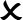	✓
Dasatinib	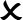	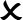	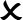	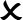	✓
Flumatinib	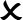	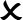	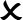	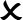	✓

## Discussion

The degree of matching between the seven cancer essential medicines listed above and the medicines recommended by the guidelines is shown in Table 8. It is evident that the degree of matching between the essential medicines for the majority of diseases and those recommended by the guidelines is low, ranging from 30 to 60%. The matching degree between the essential medicines list and the guidelines needs to be further improved.

**Table 8:** Matching degree of essential medicines list and guidelines for different diseases.

**Disease**	**Matching degree between essential** **medicines list and guidelines published in** **2018 and before**	**Matching situation**	**Matching degree between essential** **medicines list and guidelines published** **after 2018**	**Matching situation**
Liver cancer	25.00%	Extremely mismatched	16.67%	Extremely mismatched
Breast cancer	48.15%	Mismatched	57.14%	Mismatched
Esophageal cancer	63.64%	More matched	50.00%	Mismatched
Lung cancer	38.10%	Mismatched	28.57%	Extremely mismatched
Colorectal cancer	40.00%	Mismatched	30.00%	Extremely mismatched
Gastric cancer	53.85%	More matched	53.85%	More matched
Leukemia	66.67%	More matched	26.67%	More matched

The poor match of the Essential Medicines List with guidelines published in 2018 and earlier suggests that the current version of the Essential Medicines List was not constructed with sufficient attention to the guidelines. The mismatch between the essential medicines list and treatment guidelines will significantly impede the clinical medication by doctors. On the one hand, on 11 October 2019, the general office of the State Council issued opinions on further ensuring the supply and price of medicines in shortage, making it clear that the proportion of essential medicine varieties provided by primary medical and health institutions, secondary public hospitals, and tertiary public hospitals should not be <90, 80, and 60%, respectively. Therefore, hospitals must prioritize the availability of essential medicines and the clinical use of the essential medicines to satisfy the assessment requirements of essential medicines. On the other hand, considering the clinical medication effect, doctors must replace essential medicines with those with better therapeutic effects, as recommended by the guidelines. Nie Ruifang (29) reported that the lack of clinical demand is the primary reason why secondary and tertiary medical institutions lack new oncology essential medicines. Consequently, if the list of essential medicines does not match the treatment guidelines, it will be impossible to achieve the optimal clinical treatment effect under the condition of meeting the essential medicines examination standards. Therefore, we suggest that the essential medicines list should be formulated based on the therapeutic medicines recommended in treatment guidelines (8), and should include high-quality medicines and clinical needs recommended in the high-quality guidelines. Moreover, medicines recommended in the treatment guidelines but not in the essential medicines list should be considered for priority inclusion in the essential medicines list, while those in the essential medicines list but not recommended in clinical guidelines or with many clinical applications should be transferred in a timely manner. A perfect transfer in and transfer out mechanism need to be developed, and strategies to improve the matching degree between essential medicines list and clinical guidelines.

Although it was put forward in the opinions on improving the national essential medicine system (GBF [2018] No. 88) issued by the general office of the State Council on September 19, 2018, and the measures for the administration of the National Essential medicine list (Revised Draft) (hereinafter referred to as the draft) issued by the Department of drug policy and essential drug system of the National Health Commission on November 15, 2021, it has not yet been implemented. “The national essential medicine list conforms to regular evaluation and dynamic management, and the adjustment cycle should not exceed 3 years. If necessary, the adjustment can be organized in time”. However, the study indicated that the low degree of matching between various oncology essential medicines and the medicines recommended by the guidelines may be due to the lengthy update cycle of the essential medicines list. It can be seen from the results of the matching degree analysis (Table 8) that the matching degree of the list with the guidelines published after 2018 is mostly lower than the matching degree of the list with the guidelines published in 2018 and before. Medicines with superior clinical efficacy (Table 9) that have been on the market after the release of the 2018 edition of the Essential Medicines List have not been included in the Essential Medicines List, thus failing to meet clinical drug needs. In light of this, the current three-year regular adjustment and timely adjustment of the directory adjustment management mechanism needs to be further improved. When the essential medicines list is adjusted again, consideration can be given to updating the newly marketed medicines recommended by the guidelines to the list.

**Table 9:** Oncology meidicines marketed in China in 2019 and later in the above table.

**Medicine**	**Time to market**
Donafenib	June 2021
Atezolizumab	February 2020
Eribulin	July 2019
Dacomitinib	June 2019
Atezolizumab	February 2020
Tislelizumab	December 2019
Atezolizumab	February 2020
Zanubrutinib	June 2020
Orelabrutinib	December 2020
Venetoclax	December 2020
Obinutuzumab	June 2021
Flumatinib	November 2019

The above analysis showed that, in China, there are many guideline formulated by associations, such as the Chinese Medical Doctor Association and the Chinese Medical Association. In recent years, China has made great efforts in formulating and standardizing the guidelines. For example, the healthy China action (2019–2030) released in 2019 clearly mentioned the development of screening, early diagnosis and early treatment guidelines for key cancers such as gastric cancer, esophageal cancer, colorectal cancer, lung cancer, cervical cancer and breast cancer, which have a high incidence rate and mature screening methods and technical schemes, Recently, the general office of the National Health Commission issued the notice on printing and distributing the diagnosis and treatment guidelines for tumor and blood disease related diseases (2022 version), and formulated the diagnosis and treatment guidelines for 21 tumor and blood disease related diseases, including primary lung cancer and gastric cancer. In addition, the draft also proposes that the transfer in and transfer out of essential drugs should refer to the clinical diagnosis and treatment guidelines. In the future, it can further clarify how to quantify the reference guidelines when adjusting the essential medicines list, so as to improve the matching degree between the essential medicines list and the guidelines and better adapt essential mediciines to the needs of clinical medication.Therefore, we think the government should organize and establish departments to formulate scientific and authoritative standard national guidelines, preliminarily evaluate, modify, and improve the guidelines before publication (30). Such departments can also update published guidelines regularly (generally 2–5 years), to provide accurate clinical guidance (31).

In previous analyses, it was found that the essential medicines list and the treatment guidelines for diseases in China are not updated simultaneously. This implies that newly recommended medicines by the guidelines are not included in the essential medicines list. For example, the two medicines for the treatment of non-small cell lung cancer recommended by the lung cancer diagnosis and treatment guidelines, daktinib and ositinib, were approved in 2019, but they have not been in included in the essential medicines list (2018 Edition). This limits the supply of these medicines and affect patient treatment. It should be noted that the rate at which guidelines for some diseases are update is lower compared with that of the essential medicines list. Consequently, doctors still use old guidelines and prescriptions for clinical diagnosis and treatment., and the medicines newly included in the essential medicines list can not be used effectively. The lag of guideline update will affect the clinical medication of doctors and can not provide effective clinical guidance for doctors. Therefore, the national essential medicines list and other treatment guidelines should be updated simultaneously, in China. Moreover, the update cycle of the two should be shortened to comply with the changing medical field to improve consistency between guidelines and list.

The limitations of this study is that it is the first time to analyze the consistency between the essential medicines list and treatment guidelines, there is no literature to refer to the method. Therefore, there may be some disputes in the calculation of the matching degree between the essential medicines list and treatment guidelines.

## Conclusion

This is the first study to analyze the relationship between treatment guidelines and the essential medicines list in China. In addition, the quality of essential medicines was evaluated from the perspective of the consistency between the essential medicines list and the treatment guidelines for the first time. This analysis shows that, although China has made great efforts in updating and standardizing treatment guidelines in recent years, as evidenced by establishment of such as, “adjustment cycle in principle shall not exceed 3 years” and “timely adjustment”, the matching degree between oncology drugs on the national essential medicines list (2018 edition) and the oncology drugs recommended in treatment guidelines is still low. Therefore, the adjustment mechanism for essential medicines list and matching degree between the list and the guidelines should me improved to promote the utilization of essential medicines.

## Data Availability Statement

The raw data supporting the conclusions of this article will be made available by the authors, without undue reservation.

## Author Contributions

JY and LC designed the whole study and contributed to the original draft of the study. CL, XZ, and YC collected and analyzed data and took the responsibility for review and editing. All authors contributed to the article and approved the submitted version.

## Funding

This work was supported by the National Social Science Foundation of China (15ZDB167).

## Conflict of Interest

The authors declare that the research was conducted in the absence of any commercial or financial relationships that could be construed as a potential conflict of interest.

## Publisher's Note

All claims expressed in this article are solely those of the authors and do not necessarily represent those of their affiliated organizations, or those of the publisher, the editors and the reviewers. Any product that may be evaluated in this article, or claim that may be made by its manufacturer, is not guaranteed or endorsed by the publisher.
